# Suggestions for Sleeplessness

**Published:** 1879-02

**Authors:** 


SUGGESTION FOR SLEEPLESSNESS.—Many persons troubled with insomnia also suffer from cold feet. An English writer suggests that the feet be dipped in cold water for a brief period; often just to immerse them, and no more, is sufficient; and then they should be rubbed with a pair of hair flesh-gloves, or a rough Turkish towel, till they glow, immediately before getting into bed. After this, a hot-water bottle will be successful enough in maintaining the temperature of the feet, though without this preliminary, it is impotent to do so. Disagreeable as the plan at first sight may appear, it is efficient; and those who have once fairly tried it continue it, and find that they have put an end to their bad nights and cold feet. Pills, potions, lozenges, "night-caps," all narcotics, fail to enable the sufferer to woo sleep successfully; get rid of the cold feet, and then sleep will come of itself.—*Medical and Surgical Reporter.*

